# A RESTful API for Accessing Microbial Community Data for MG-RAST

**DOI:** 10.1371/journal.pcbi.1004008

**Published:** 2015-01-08

**Authors:** Andreas Wilke, Jared Bischof, Travis Harrison, Tom Brettin, Mark D'Souza, Wolfgang Gerlach, Hunter Matthews, Tobias Paczian, Jared Wilkening, Elizabeth M. Glass, Narayan Desai, Folker Meyer

**Affiliations:** 1 Mathematics and Computer Science Division, Argonne National Laboratory, Lemont, Illinois, United States of America; 2 Computation Institute, University of Chicago, Chicago, Illinois, United States of America; University of Canterbury, New Zealand

## Abstract

Metagenomic sequencing has produced significant amounts of data in recent years. For example, as of summer 2013, MG-RAST has been used to annotate over 110,000 data sets totaling over 43 Terabases. With metagenomic sequencing finding even wider adoption in the scientific community, the existing web-based analysis tools and infrastructure in MG-RAST provide limited capability for data retrieval and analysis, such as comparative analysis between multiple data sets. Moreover, although the system provides many analysis tools, it is not comprehensive. By opening MG-RAST up via a web services API (application programmers interface) we have greatly expanded access to MG-RAST data, as well as provided a mechanism for the use of third-party analysis tools with MG-RAST data. This RESTful API makes all data and data objects created by the MG-RAST pipeline accessible as JSON objects. As part of the DOE Systems Biology Knowledgebase project (KBase, http://kbase.us) we have implemented a web services API for MG-RAST. This API complements the existing MG-RAST web interface and constitutes the basis of KBase's microbial community capabilities. In addition, the API exposes a comprehensive collection of data to programmers. This API, which uses a RESTful (Representational State Transfer) implementation, is compatible with most programming environments and should be easy to use for end users and third parties. It provides comprehensive access to sequence data, quality control results, annotations, and many other data types. Where feasible, we have used standards to expose data and metadata. Code examples are provided in a number of languages both to show the versatility of the API and to provide a starting point for users. We present an API that exposes the data in MG-RAST for consumption by our users, greatly enhancing the utility of the MG-RAST service.

## Author Summary

Metagenomic sequencing has produced significant amounts of data in recent years. For example, as of summer 2013, the MG-RAST metagenomics analysis system has been used to annotate over 110,000 data sets totaling over 43 Terabases. With metagenomic sequencing finding even wider adoption in the scientific community, the existing web-based analysis tools and infrastructure in MG-RAST provide limited capability for comparative analysis (i.e., number of data sets). Moreover, although the system provides many analysis tools, it is not comprehensive. By opening MG-RAST up via a web services API (application programmers interface) we have enabled a programmatic way for others to use their bioinformatics tools with MG-RAST data.

This is a *PLOS Computational Biology* Software Article.

## Introduction

Over 110,000 metagenomic data sets have been uploaded and analyzed in MG-RAST [1] since 2007, totaling over 43 Terabases (TBp). Data uploaded falls in three classes: shotgun metagenomic data, amplicon data, and, more recently, metatranscriptomic data. The MG-RAST pipeline normalizes all samples by applying a uniform pipeline with the appropriate quality control mechanisms for the various data sources. Uniform processing and robust sequence quality control enable comparison across experimental systems and, to some extent, across sequencing platforms. With the inclusion of standardized metadata [2] MG-RAST has enabled meta-analysis available through its web-based user interface at http://metagenomics.anl.gov. The user interface provides an easy-to-use way to upload data access data via download or interface, perform analyses, and create and share projects. As with most GUIs, however, there are limitations to what can be done. Examples of this include the number of samples processed in a single analysis, access to complete metadata, and easy access to raw data and quality metrics for each sample.

As part of the DOE Systems Biology Knowledgebase project (KBase) we have implemented a web services application programmers interface (API) that exposes all data to (authenticated) programmers, enabling users to access available data and functionality through software applications. User access to MG-RAST's internal data structures is now possible.

## Design and Implementation

The MG-RAST API enables programmatic access to data and analyses in MG-RAST without requiring local installations. With the new API, users can authenticate against the service, submit their data, download results, and perform extensive comparisons of data sets. We chose to use the Representational State Transfer (REST) [3] architecture. The REST approach allows download of data in ASCII format, which allows users to query the system via URLs and returns MG-RAST data objects in their native format (e.g. similarity tables or sequence files). For structured data (e.g. metadata or project information) the MG-RAST API uses JSON (Javascript Object Notation, a widely used standard) as its data format.

Using this approach users can use simple tools to download data files to their machines or view the JSON in their web browsers using one of the many available JSON viewers. In addition, many programming languages have libraries for convenient HTTP interaction and JSON conversions.

This article focuses on describing the architecture used - the underlying components of a web services architecture, their interactions, and the data used for their operation. REST has several key advantages for system scalability. Unlike more traditional remote procedure call methods, REST APIs make the semantics of requests visible at the HTTP protocol layer. This makes the system easier to scale, optimize, and harden through the use of HTTP level appliances providing security, caching, and proxy capabilities. REST APIs also have useful properties in terms of client adoption. They have a minimal number of prerequisites and any language with HTTP and JSON support or command line utilities, such as "curl", can easily integrate with the design.

The MG-RAST RESTful API supports introspection and versioning. In order to access a specific version of the API, the version number must be added to the base URL. The base URL for all API calls is http://api.metagenomics.anl.gov. Calling the base URL of the API without any options returns a list and description of available resources; calling a resource without any options returns a description of the resource and its request options with example calls.

**Figure 1:**
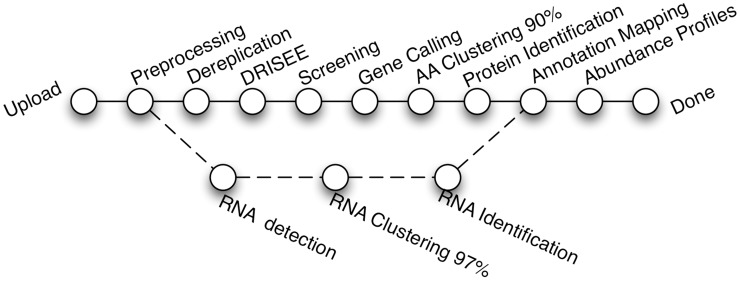
Different stages of the MG-RAST automated pipeline. In the annotation mapping stage, functions and taxonomic units from the M5nr are mapped to the MD5 identifiers found in the similarity search.

The MG-RAST pipeline accepts sequences in a variety of formats from most DNA sequencing platforms and transforms all sequences using automated pipelines (see Figure 1). The pipeline performs quality control, protein prediction, clustering, and similarity-based annotation on nucleic acid sequence data sets.

The analyses provided by MG-RAST rely, to some extent, on comparison with external protein databases, maintained as a single data product in the M5nr [4], and enabling users to switch annotation sources and thus naming conventions used for annotation at analysis time. Using the M5nr database, MG-RAST provides links to all major sequence databases and, for example, allows linking from metagenomic sequences to complete genomes (see Table 1 for a list of available namespaces).

**Table 1:** Current annotation sources available in MG-RAST via the M5nr mechanism.

Database	Source	Type	#IDs
GenBank	NCBIhttp://www.ncbi.nlm.nih.gov/	protein	20,977,345
IMG	JGIhttp://img.jgi.doe.gov/	protein	11,306,919
InterPro	EBIhttp://www.ebi.ac.uk/interpro	protein	22,666
KEGG	KEGGhttp://www.genome.jp/kegg	protein	6,071,792
PATRIC	VBIhttp://www.patricbrc.org/	protein	13,612,238
Phantome	Phantomehttp://www.phantome.org/	protein	67,876
RefSeq	NCBIhttp://www.ncbi.nlm.nih.gov/	protein	14,875,735
SEED	SEEDhttp://www.theseed.org/	protein	15,822,645
SwissProt	UniProthttp://www.uniprot.org/	protein	535,248
TrEMBL	UniProthttp://www.uniprot.org/	protein	20,639,311
COG	eggNOGhttp://eggnog.embl.de/	functional hierarchy	7,321
GO	GOhttp://www.geneontology.org/	functional hierarchy	19,849
KO	KEGGhttp://www.genome.jp/kegg	functional hierarchy	13,584
NOG	eggNOGhttp://eggnog.embl.de/	functional hierarchy	37,941
Subsystems	SEEDhttp://www.theseed.org/	functional hierarchy	13,912

Users are provided access to these MG-RAST resources as well as to analysis results being produced (public data and the users' own data). Table 2 lists the high-level objects that can be accessed; in addition, users can upload sequence and metadata into their own private MG-RAST staging area. Some objects (e.g., metagenome, metadata, project, M5nr database) will seem intuitive, while others are different from what most users would expect (e.g., download, annotation, matrix). We have designed these additional objects to allow rapid access to sets of sequences or analysis results related for a data set (download), annotated sequences or BLAT results for a data set (annotation), and abundance information for many data sets (matrix).

**Table 2:** Top-level resources available through the MG-RAST-API.

Resource/Object	Description
annotation	Taxonomic and functional annotations made by comparison with the M5nr database.
compute	Resource to compute PCoA, heatmap, and normalization for a set of input metagenomes.
download	Download results of the MG-RAST pipeline.
inbox	Upload and listing of data in the staging area prior to pipeline execution.
library	Library information for uploaded metagenome provided by the user.
matrix	Abundance profiles in BIOM (5) format for a list of metagenomes.
M5nr	Access M5 nonredundant protein database used for sequence annotation.
metadata	Creation, export, and validation of metadata templates and spreadsheets.
metagenome	Container for sample, library, project, and precomputed data for an uploaded metagenomic sequence file.
project	Project summary for metagenome provided by user
sample	Sample information provided by user
search	Search MG-RAST by MG-ID, metadata, function, or taxonomy; or implement a more complex search.

Most of the API calls are simply URLs, which can be entered in the address bar of a web browser to perform the download through the browser. These URLs can also be used with a command line tool like curl, in programing-language-specific libraries, or in command line scripts. The examples in the Results section illustrate the use of each of these methods. The example scripts are available on in the supplementary materials and on GitHub (https://github.com/MG-RAST/MG-RAST-Tools) along with other useful illustrative scripts.

## Results

The MG-RAST API provides unprecedented access to MG-RAST data. The system provides index- driven access to data subsets using the following data types as indices into the data: functions, functional hierarchy data, and taxonomic data. Whenever possible we have employed standards to expose data and metadata, such as the BIOM [5] standard for encoding abundance profiles.

Next, we demonstrate a number of straightforward use cases for the more traditional objects.

### Annotation

MG-RAST enables users to extract data based on functional or taxonomic annotations. The necessary functionality is provided by two API calls. The first API (Box 1) call lists all metagenomes with certain metadata fields and functional contents, the second API call extracts all requested reads from a given metagenome. The following example script exploits these two API calls to produce a file with sequences annotated as proteases, using SEED annotations from all samples from marine environments. The reads are labeled with the originating data set and the read identifier, as well as the underlying similarity result.

**Box 1. Example API Call for Search and Data Retrieval**

API base URL: http://api.metagenomics.anl.gov

API version: 1

Resources: metagenome and annotation

Example API calls:

 1. Find all marine metagenomes with reads annotated as protease: http://api.metagenomics.anl.gov/1/metagenome?biome=marine&function=protease

 2. Retrieve all reads from a marine metagenome (here mgm4440036.3) annotated as protease in SEED Subsystems: http://api.metagenomics.anl.gov/1/annotation/sequence/mgm4440036.3?type=function&filter=protease&source=Subsystems

Example command line script combining the two calls: mg-extract-sequences.py —function "protease" —biome "marine" | head -n 6

Example output^2^:

sequence id m5nr id (md5sum) semicolon separated list of annotations dna sequence

mgm4440036.3|5079086 02f5a5ecb261e0d3684bbfa2e4e8eb2f ATP-dependent hsl protease ATP-binding subunit HslU AGAAGACTTTCTAGATTAGCTGAGGCACCTTTCATAAAAGTTGAAGCAACAAGATTTACTGAGGTTGGGTACGTAGGTAGAGATGTTGAACAAATAGTGAGAGATTTG

mgm4440036.3|5096297 06dd4b98b30c90b9d057ae77500e7a37 ATP-dependent hsl protease ATP-binding subunit HslU ATTTTCAACTGATGCATTTACTTCTGCAGATATTTTTGCAAGCATCTCAATACCGTCGTCGGTAAACTCCAATGCTACGTTTTCTGTGTTTAGCAAAGCTTTA

mgm4440036.3|5111934 06dd4b98b30c90b9d057ae77500e7a37 ATP-dependent hsl protease ATP-binding subunit HslU ATTTCAACTGATGCATTTACTTCTGCAGATATTTTTGCAAGCATCTCAATACCGTCGTCGGTAAACTCCAATGCTACGTTTTCTGTGTTTAAGCAAAGCTTTA

### Download

Download allows users to extract analysis result files from MG-RAST (Box 2). The following example below shows how to download BLAT [6] results for a given metagenome.

**Box 2. Example API Call for Downloading Similarities File**

API base URL: http://api.metagenomics.anl.gov

API version: 1

Resources: download

Example command line call using curl to download the similarities file for metagenome mgm4447943.3:

curl " http://api.metagenomics.anl.gov/1/download/mgm4447943.3?file=650.1 "> mgm4447943.3.sims.gz

Example output:

GF8803K01A004I_1_134_- 3a215838fa48c7b3d66bc0f406273927 93.33 30 2 0 10 39 191 220 2.6e-09 60.0

GF8803K01A004I_1_134_- 1233890f05d5697e10a58d1dcfdd8c5c 86.67 30 4 0 10 39 191 220 1.7e-08 57.0

GF8803K01A004I_1_134_- 1f79a21185584c67236c773221a1783f 86.67 30 4 0 10 39 191 220 1.7e-08 57.0

GF8803K01A004I_1_134_- 1a8348be8fb06a58583961d5988da4b6 86.67 30 4 0 10 39 178 207 3.9e-08 56.0

GF8803K01A004I_1_134_- ed70df8837c6379d80a4b714d6be3cbf 83.87 31 5 0 10 40 192 222 5.1e-08 56.0

GF8803K01A004I_1_134_- f22757e1b3c4bd87799cc53659a9337c 86.67 30 4 0 10 39 192 221 3.9e-08 56.0

GF8803K01A004I_1_134_- 119ae93780cf45093da9718a3a89c77e 86.67 30 4 0 10 39 191 220 8.8e-08 55.0

GF8803K01A004I_1_134_- bec613da1370240331eed0c3a12033f7 86.67 30 4 0 10 39 192 221 6.7e-08 55.0

### Inbox

The inbox is a staging area where users can upload metadata and sequence files and manage their data. This requires a MG-RAST account and user authentication (Box 3). An authentication token can be created through the user preferences in MG-RAST.

**Box 3. Example API Call to Upload the File 'sequences.fastq' to User Inbox, auth Is Required, the *auth_key* Can Be Obtained through User Preferences in MG-RAST**

curl -X POST -H "auth: auth_key" -F "upload = @sequences.fastq" " http://api.metagenomics.anl.gov/1/inbox "

### Matrix

Users can retrieve abundance profiles (Box 4) based on functional or taxonomic profiles. Default output format is BIOM.

**Box 4. Example API Call for Retrieving Taxonomic Abundances for SEED on Family Level for 2 Metagenomes**

http://api.metagenomics.anl.gov/matrix/organism?group_level=family&source=SEED&evalue=5&id=mgm4440442.5&id=mgm4440026.3

### M5nr

As mentioned earlier, we use a M5-based nonredundant database to perform annotations. Here is an example of extracting the UniProt database entry record for a given sequence in a metagenome (Box 5). Using the M5nr, we identify the UniProt database record most similar to the sequence of a given feature.

**Box 5. Example API Calls to Retrieve a Uniprot Entry Using the M5nr and Uniprot API**

1. Retrieve the UniProt ID for a given sequence identifier: http://api.metagenomics.anl.gov/1/m5nr/md5/ffc62262a18b38671c3e337150ef535f?source=SwissProt
2. Use the UniProt API to access the database record: http://www.uniprot.org/uniprot/B8DWI2.txt

Example command line call:

mg-retrieve-uniprot.py —md5 ffc62262a18b38671c3e337150ef535f —source SwissProt

Example output:

ID DAPA_BIFA0 Reviewed; 303 AA.

AC B8DWI2;

DT 28-JUL-2009, integrated into UniProtKB/Swiss-Prot.

DT 03-MAR-2009, sequence version 1.

DT 16-OCT-2013, entry version 34.

DE RecName: Full = 4-hydroxy-tetrahydrodipicolinate synthase;

DE Short = HTPA synthase;

DE EC = 4.3.3.7;

GN Name = dapA; OrderedLocusNames = BLA_0534;

OS Bifidobacterium animalis subsp. lactis (strain AD011).

#### Metagenome

Users can access a metagenome by its ID, such as mgm4440026.3, from the command line as shown in Box 6.

**Box 6. Example API Line Call for the Metadata for a Metagenome**

curl " http://api.metagenomics.anl.gov/1/metagenome/mgm4440026.3 " | json_xs

{

"version": 1,

"project": [

"mgp31",

" http://api.metagenomics.anl.gov/1/project/mgp31 "

],

"status": "public",

"name": "CFLungPat001Rep1SDVir20060505",

"sequence_type": "WGS",

"library": [

"mgl43388",

" http://api.metagenomics.anl.gov/1/library/mgl43388 "

],

"created": "2007-04-27 14:47:11",

"url": " http://api.metagenomics.anl.gov/1/metagenome/mgm4440026.3?verbosity=minimal ",

"id": "mgm4440026.3",

"sample": [

"mgs12326",

" http://api.metagenomics.anl.gov/1/sample/mgs12326 "

]

}

MG-RAST enables users to directly retrieve sample, library, and project information, allowing different granularity of the data being retrieved.

### Project

Users can retrieve project information (Box 7) by using project ID and output as a JSON formatted file.

**Box 7. Example of Retrieving Project Information**

curl " http://api.metagenomics.anl.gov/project/mgp31?verbosity=full " | json_xs

### Sample

Available information about individual samples, including IDs and metadata, can be accessed as shown in Box 8.

**Box 8. Example API Call to Retrieve Sample Information from a Metagenome**

http://api.metagenomics.anl.gov/1/sample/mgs12326?verbosity=full

### Search

Using the search resource, users can search for data they want to retrieve. Queries can be made for, metadata, function, and taxonomy (Box 9). Complex queries are supported.

**Box 9. Complex Search Using Function, Metadata, and Taxonomy**

Example API call:

http://api.metagenomics.anl.gov/metagenome?function=dnaA&organism=coli&biome=marine&match=all&order=created

### Access control allows access to private and public data

In MG-RAST, all data is initially private. Users who submit data can decide to share that data with specific users (by typing in an email address for the users) or make the data publicly available. Both actions require the provision of standard-compliant minimal metadata by the submitting user. The API provides access to both public and nonpublic data, requiring users to submit authentication tokens for access to private data.

Authentication tokens can be obtained via the MG-RAST web interface through the user preferences page and are valid for up to 14 days (Box 10). The token serves as login and password for the API. Below is an example of how to use the tokens in three different scenarios. Users can invalidate a token at any time by generating a new one. Note that accessing a remote site through an XMHttpRequest requires support for Cross-Origin Resource Sharing (CORS) compliance and Preflight Request. CORS requires the remote site to accept the local site's origin (AccessControl AllowOrigin). For Preflight Requests, if an HTTP request from a browser adds a custom header to the request (in the example “AUTH”), the browser first makes an OPTIONS request to the largest server, inquiring whether AccessControlAllowHeaders allows this header and whether AccessControlAllowMethod allows the request method (GET/POST).

**Box 10. Example Authentication Script**

PERL

access_control.pl

# get a user agent

my $ua  =  LWP::UserAgent->new;

# set the authentication header

$ua->default_header('AUTH'  = > $auth_token);

# retrieve data requiring authentication

print $ua->get(" http://api.metagenomics.anl.gov/metagenome/mgm12345.3 ")->content;

curl

curl -s -X GET -H "AUTH: myAuthTokenHere" " http://api.metagenomics.anl.gov/metagenome/mgm12345.3 "

javascript

var xhr  =  new XMLHttpRequest();

xhr.open('GET'," http://api.metagenomics.anl.gov/metagenome/mgm12345.3 ");

xhr.setRequestHeader('AUTH', auth_token);

xhr.onload  =  function() {var metagenome  =  JSON.parse(xhr.responseText);}

## Availability and Future Directions

RESTful web services provide access to the data products in MG-RAST. While MG-RAST is open source (GitHub), third parties who are interested in the comparative analysis provided by MG-RAST must either download and install all analysis products or (worse) repeat the analysis. In a time of rapidly decreasing data generation cost [7] and rising data analysis cost [8], both reanalysis and local transfer of data products do not appear to be viable options, while the RESTful web services do.

We anticipate that with the provision of these comprehensive web services, a significant number of users will create their own of data pipelines feeding into or reading from MG-RAST.

While the MG-RAST pipeline must be optimized with each release in order to keep pace with the growing body of sequence data (within one major release, we do not alter the pipeline other than bug fixes), many uses cases are conceivable that apply more computationally expensive approaches. For example, we may want to determine protein subfamily assignments using more expensive algorithms such as Pfam [9], InterPro [10], or FAT-CAT [11]) or other approaches for mapping metagenomic protein fragments to (precomputed) trees. We note that the relatively noisy nature of most shotgun metagenomic data (with or without assembly) casts some doubt over many sequence analyses that attempt to extract weak signals from the sequences. The rare biosphere debate [12] has demonstrated that without the use of denoising techniques or accounting for the noise in the sequence data, diversity estimates for amplicon samples will be inflated. The data-set-wide quality estimates computed by MG-RAST [13] allow users to exclude certain data sets in their (meta)analyses.

Although using a web services interface lightens the installation burden, having to transfer the data across the Internet creates significant overhead, which presents a drawback. In analyzing usage patterns, however, we have found that typically only a small subset of the computed data for each data set is actually accessed by most users. The organization of the data and the data products reflects that pattern. The abundance profiles summarize abundance of taxa, functions (in various namespaces), and gene (protein) functional hierarchies while retaining the ability for the user to set cutoffs (e-value, alignment length, and percent identity). This structure enables decision making in the presentation layer by users or providers of web interfaces. Together with index-supported subset retrieval, this provides the tools needed by most users to perform their comparisons and drill down to their area of choice.

Thus, from an end-user perspective, the overhead caused by the Internet transfer is more than compensated by the "filtering" that can be performed by MG-RAST. Frequently, this makes the upload of the metagenomic sequence data the single biggest data transfer, since the downloads for the abundance profile are significantly smaller than the uploaded sequences. This is an interesting observation since the various data analysis steps add to the volume of the data: typically the on-disk footprint is about 10 times the size of the uploaded sequence data.

We have chosen JSON [14] for encoding for the data because it is the current default solution for web service interfaces. This differs from the API to the SEED genome servers [15], which implements a Remote Procedure Call (RPC)-style interface. The style we chose, however, is a better fit for the data and volumes being handled by MG-RAST. In addition, it allows caching and proxying, providing additional flexibility for future solutions implemented on top of the MG-RAST API.

The RESTful API adds significant value by enabling user-generated code to access MG-RAST analyses. We have provided, in this article and on the API home page, multiple examples that show the new functionality that we have added to the existing MG-RAST service. In addition, we are creating a repository of user-contributed scripts that utilize MG-RAST as part of the contrib branch in the MG-RAST GitHub repository (https://github.com/MG-RAST/MG-RAST-Tools).

As with the companion API for the SEED-based RAST and SEED web sites, we expect a significant number of users to access the growing number of metagenomes and analysis results stored in MG-RAST using the new MG-RAST API.

Finally, the DOE Systems Biology Knowledgebase (KBase; http://kbase.us) has adopted the MG- RAST pipeline as its first automated analysis workflow for microbial community data. A version of this API is available to access microbial community data from within KBase.

### Availability and requirements

- **Project name**: MG-RAST REST API
- **Project home page**: http://api.metagenomics.anl.gov/api.html
- **Project source**: https://github.com/MG-RAST/MG-RASThttps://github.com/MG-RAST/MG-RAST https://github.com/MG-RAST/MG-RAST-Tools
- **Open Source License**: MG-RAST is made available under a BSD type LICENSE.
- **Operating system(s)**: Platform independent
- **Programming language**: Language independent
- **Other requirements**: none
- **Any restrictions to use by non-academics**: no limitations

## Supporting Information

S1 Example A full-length example and abbreviated output for searching MG-RAST for function and sequence.
(DOCX)

S2 Example A full-length example and abbreviated output for downloading data.
(DOCX)

S3 Example A full-length example and abbreviated output for data in the staging area prior to pipeline execution (Inbox).
(DOCX)

S4 Example A full-length example and abbreviated output for retrieving abundance profiles in BIOM format for a list of metagenomes (Matrix).
(DOCX)

S5 Example A full-length example and abbreviated output for etrieve M5nr annotation by source.
(DOCX)

S6 Example A full-length example and abbreviated output for retrieving sample information by metagenome ID.
(DOCX)

S7 Example A full-length example and abbreviated output for retrieving associated sample information by project ID.
(DOCX)

S8 Example A full-length example and abbreviated output for retrieving sample information.
(DOCX)

S9 Example A full-length example and abbreviated output for searching by metagenome ID.
(DOCX)

S10 Example A full-length example and abbreviated output for searching metagenomes by metadata.
(DOCX)

S11 Example A full-length example and abbreviated output for searching by function.
(DOCX)

S12 Example A full-length example and abbreviated output for a complex search using function, metadata, and taxonomy.
(DOCX)

S13 Example Access control.
(DOCX)

S14 Example An example of metagenome as JSON object.
(DOCX)

S1 Script An example python script that will retrieve annotated sequences from metagenomes filtered by function name and metadata.
(PY) Click here for additional data file.

S2 Script An example python script that will retrieve the Uniprot result for a sequence md5 or accession id from MG-RAST.
(PY) Click here for additional data file.

S1 File Instructions on how to invoke the example python scripts.
(TXT)
