# Supplementary material for: A RESTful API for Accessing Microbial Community Data for MG-RAST
Source: PLoS Comput Biol. 2015 Jan 8;11(1):e1004008. doi: 10.1371/journal.pcbi.1004008 (PMC4287624; doi:10.1371/journal.pcbi.1004008)
Supplement: S1 Example — A full-length example and abbreviated output for searching MG-RAST for function and sequence. (DOCX) [file pcbi.1004008.s001.docx]

API calls:

1. Find all marine metagenomes with reads annotated as protease:
   http://api.metagenomics.anl.gov/1/metagenome?biome=marine&function=protease
2. Retrieve all reads from a marine metagenome (here mgm4440036.3) annotated as protease in SEED Subsystems: http://api.metagenomics.anl.gov/1/annotation/sequence/mgm4440036.3?type=function&filter=protease&source=Subsystems

Example command line script combining the two calls:

mg-extract-sequences.py --function "protease" --biome "marine" | head -n 6

Example output:

sequence id m5nr id (md5sum) semicolon separated list of annotations dna sequence

mgm4440036.3|5079086 02f5a5ecb261e0d3684bbfa2e4e8eb2f ATP-dependent hsl protease ATP-binding subunit HslU AGAAGACTTTCTAGATTAGCTGAGGCACCTTTCATAAAAGTTGAAGCAACAAGATTTACTGAGGTTGGGTACGTAGGTAGAGATGTTGAACAAATAGTGAGAGATTTG

mgm4440036.3|5096297 06dd4b98b30c90b9d057ae77500e7a37 ATP-dependent hsl protease ATP-binding subunit HslU ATTTTCAACTGATGCATTTACTTCTGCAGATATTTTTGCAAGCATCTCAATACCGTCGTCGGTAAACTCCAATGCTACGTTTTCTGTGTTTAGCAAAGCTTTA

mgm4440036.3|5111934 06dd4b98b30c90b9d057ae77500e7a37 ATP-dependent hsl protease ATP-binding subunit HslU ATTTCAACTGATGCATTTACTTCTGCAGATATTTTTGCAAGCATCTCAATACCGTCGTCGGTAAACTCCAATGCTACGTTTTCTGTGTTTAAGCAAAGCTTTA
