# Supplementary material for: A RESTful API for Accessing Microbial Community Data for MG-RAST
Source: PLoS Comput Biol. 2015 Jan 8;11(1):e1004008. doi: 10.1371/journal.pcbi.1004008 (PMC4287624; doi:10.1371/journal.pcbi.1004008)
Supplement: S2 Example — A full-length example and abbreviated output for downloading data. (DOCX) [file pcbi.1004008.s002.docx]

Example cmd-line:

curl "http://api.metagenomics.anl.gov/download/mgm4447943.3?file=650.1" > mgm4447943.3.sims.gz

Example output:

GF8803K01A004I_1_134_- 3a215838fa48c7b3d66bc0f406273927 93.33 30 2 0 10 39 191 220 2.6e-09 60.0

GF8803K01A004I_1_134_- 1233890f05d5697e10a58d1dcfdd8c5c 86.67 30 4 0 10 39 191 220 1.7e-08 57.0

GF8803K01A004I_1_134_- 1f79a21185584c67236c773221a1783f 86.67 30 4 0 10 39 191 220 1.7e-08 57.0

GF8803K01A004I_1_134_- 1a8348be8fb06a58583961d5988da4b6 86.67 30 4 0 10 39 178 207 3.9e-08 56.0

GF8803K01A004I_1_134_- ed70df8837c6379d80a4b714d6be3cbf 83.87 31 5 0 10 40 192 222 5.1e-08 56.0

GF8803K01A004I_1_134_- f22757e1b3c4bd87799cc53659a9337c 86.67 30 4 0 10 39 192 221 3.9e-08 56.0

GF8803K01A004I_1_134_- 119ae93780cf45093da9718a3a89c77e 86.67 30 4 0 10 39 191 220 8.8e-08 55.0

GF8803K01A004I_1_134_- bec613da1370240331eed0c3a12033f7 86.67 30 4 0 10 39 192 221 6.7e-08 55.0
