# Supplementary material for: A RESTful API for Accessing Microbial Community Data for MG-RAST
Source: PLoS Comput Biol. 2015 Jan 8;11(1):e1004008. doi: 10.1371/journal.pcbi.1004008 (PMC4287624; doi:10.1371/journal.pcbi.1004008)
Supplement: S3 Example — A full-length example and abbreviated output for data in the staging area prior to pipeline execution (Inbox). (DOCX) [file pcbi.1004008.s003.docx]

Upload file 'sequences.fastq' to user inbox, auth is required, the ***auth_key*** can be obtained through user preferences in MG-RAST

curl -X POST -H "auth: auth_key" -F "upload=@sequences.fastq" "http://api.metagenomics.anl.gov/1/inbox"
