# Supplementary material for: A RESTful API for Accessing Microbial Community Data for MG-RAST
Source: PLoS Comput Biol. 2015 Jan 8;11(1):e1004008. doi: 10.1371/journal.pcbi.1004008 (PMC4287624; doi:10.1371/journal.pcbi.1004008)
Supplement: S4 Example — A full-length example and abbreviated output for retrieving abundance profiles in BIOM format for a list of metagenomes (Matrix). (DOCX) [file pcbi.1004008.s004.docx]

API call:

http://api.metagenomics.anl.gov/matrix/organism?group_level=family&source=SEED&evalue=5&id=mgm4440442.5&id=mgm4440026.3

Example cmd-line:

mg-compare-taxa.py --ids mgm4440442.5,mgm4440026.3 --level family --source SEED --evalue 5 --format biom | json_xs | head -30

Example output:

{

"columns": [

{

"id": "mgm4440026.3",

"metadata": null,

"name": "CFLungPat001Rep1SDVir20060505"

},

{

"id": "mgm4440442.5",

"metadata": null,

"name": "HealSputRep2SDVir20060707"

}

],

"data": [

[

0,

1,

3

],

[

1,

1,

1

],

[

2,

1,

1

],
