# Supplementary material for: A RESTful API for Accessing Microbial Community Data for MG-RAST
Source: PLoS Comput Biol. 2015 Jan 8;11(1):e1004008. doi: 10.1371/journal.pcbi.1004008 (PMC4287624; doi:10.1371/journal.pcbi.1004008)
Supplement: S5 Example — A full-length example and abbreviated output for etrieve M5nr annotation by source. (DOCX) [file pcbi.1004008.s005.docx]

API calls:

1.http://api.metagenomics.anl.gov/1/m5nr/md5/ffc62262a18b38671c3e337150ef535f?source=SwissProt

2. http://www.uniprot.org/uniprot/B8DWI2.txt

Example cmd-line:

mg-retrieve-uniprot.py --md5 ffc62262a18b38671c3e337150ef535f --source SwissProt

Example output:

ID DAPA_BIFA0 Reviewed; 303 AA.

AC B8DWI2;

DT 28-JUL-2009, integrated into UniProtKB/Swiss-Prot.

DT 03-MAR-2009, sequence version 1.

DT 16-OCT-2013, entry version 34.

DE RecName: Full=4-hydroxy-tetrahydrodipicolinate synthase;

DE Short=HTPA synthase;

DE EC=4.3.3.7;

GN Name=dapA; OrderedLocusNames=BLA_0534;

OS Bifidobacterium animalis subsp. lactis (strain AD011).
