# Supplementary material for: A RESTful API for Accessing Microbial Community Data for MG-RAST
Source: PLoS Comput Biol. 2015 Jan 8;11(1):e1004008. doi: 10.1371/journal.pcbi.1004008 (PMC4287624; doi:10.1371/journal.pcbi.1004008)
Supplement: S6 Example — A full-length example and abbreviated output for retrieving sample information by metagenome ID. (DOCX) [file pcbi.1004008.s006.docx]

curl "http://api.metagenomics.anl.gov/1/metagenome/mgm4440026.3" | json_xs

{

"version": 1,

"project": [

"mgp31",

"http://api.metagenomics.anl.gov/1/project/mgp31"

],

"status": "public",

"name": "CFLungPat001Rep1SDVir20060505",

"sequence_type": "WGS",

"library": [

"mgl43388",

"http://api.metagenomics.anl.gov/1/library/mgl43388"

],

"created": "2007-04-27 14:47:11",

"url": "http://api.metagenomics.anl.gov/1/metagenome/mgm4440026.3?verbosity=minimal",

"id": "mgm4440026.3",

"sample": [

"mgs12326",

"http://api.metagenomics.anl.gov/1/sample/mgs12326"

]

}
