# Supplementary material for: A RESTful API for Accessing Microbial Community Data for MG-RAST
Source: PLoS Comput Biol. 2015 Jan 8;11(1):e1004008. doi: 10.1371/journal.pcbi.1004008 (PMC4287624; doi:10.1371/journal.pcbi.1004008)
Supplement: S7 Example — A full-length example and abbreviated output for retrieving associated sample information by project ID. (DOCX) [file pcbi.1004008.s007.docx]

curl "http://api.metagenomics.anl.gov/project/mgp31?verbosity=full" | json_xs

Output:

{

"status" : "public",

"version" : 1,

"name" : "Human Lung Healthy vs Cystic Fibrosis Metagenome",

"metagenomes" : [

[

"mgm4440442.4",

"http://api.metagenomics.anl.gov/1/metagenome/mgm4440442.4"

],

[

"mgm4440442.5",

"http://api.metagenomics.anl.gov/1/metagenome/mgm4440442.5"

],

[

"mgm4440026.3",

"http://api.metagenomics.anl.gov/1/metagenome/mgm4440026.3"

],

[

"mgm4440051.3",

"http://api.metagenomics.anl.gov/1/metagenome/mgm4440051.3"

],

[

"mgm4440441.3",

"http://api.metagenomics.anl.gov/1/metagenome/mgm4440441.3"

]

],

"libraries" : [

[

"mgl42984",

"http://api.metagenomics.anl.gov/1/library/mgl42984"

],

[

"mgl43186",

"http://api.metagenomics.anl.gov/1/library/mgl43186"

],

[

"mgl43388",

"http://api.metagenomics.anl.gov/1/library/mgl43388"

],

[

"mgl43592",

"http://api.metagenomics.anl.gov/1/library/mgl43592"

],

[

"mgl43794",

"http://api.metagenomics.anl.gov/1/library/mgl43794"

]

],

"description" : "The human respiratory tract is constantly exposed to a wide variety of viruses, microbes and inorganic particulates from environmental air, water and food. Physical characteristics of inhaled particles and airway mucosal immunity determine which viruses and microbes will persist in the airways. Here we present the first metagenomic study of DNA viral communities in the airways of diseased and non-diseased individuals. We obtained sequences from sputum DNA viral communities in 5 individuals with cystic fibrosis (CF) and 5 individuals without the disease.These results have important clinical implications for CF, indicating that therapeutic measures may be more effective if used to change the respiratory environment, as opposed to shifting the taxonomic composition of resident microbiota.",

"created" : "",

"samples" : [

[

"mgs12104",

"http://api.metagenomics.anl.gov/1/sample/mgs12104"

],

[

"mgs12215",

"http://api.metagenomics.anl.gov/1/sample/mgs12215"

],

[

"mgs12326",

"http://api.metagenomics.anl.gov/1/sample/mgs12326"

],

[

"mgs12438",

"http://api.metagenomics.anl.gov/1/sample/mgs12438"

],

[

"mgs12549",

"http://api.metagenomics.anl.gov/1/sample/mgs12549"

]

],

"funding_source" : "Cystic Foundation Research Inc. (www.cfri.org) ",

"url" : "http://api.metagenomics.anl.gov/1/project/mgp31",

"pi" : "Dana Willner",

"id" : "mgp31",

"metadata" : {

"firstname" : " - ",

"PI_firstname" : "Dana",

"PI_organization_url" : "http://sdsu.edu",

"lastname" : " - ",

"organization_url" : " - ",
