# Supplementary material for: A RESTful API for Accessing Microbial Community Data for MG-RAST
Source: PLoS Comput Biol. 2015 Jan 8;11(1):e1004008. doi: 10.1371/journal.pcbi.1004008 (PMC4287624; doi:10.1371/journal.pcbi.1004008)
Supplement: S8 Example — A full-length example and abbreviated output for retrieving sample information. (DOCX) [file pcbi.1004008.s008.docx]

API call:

[http://api.metagenomics.anl.gov/1/sample/mgs12326?verbosity=fullhttp://api.metagenomics.anl.gov/1/sample/mgs12326?verbosity=full](http://api.metagenomics.anl.gov/1/sample/mgs12326?verbosity=full)

<http://api.metagenomics.anl.gov/1/sample/mgs12326?verbosity=full>

Output:

{

"project": [

"mgp31",

"http://api.metagenomics.anl.gov/1/project/mgp31"

],

"version": 1,

"name": "mgs12326",

"metagenomes": [

[

"mgm4440026.3",

"http://api.metagenomics.anl.gov/1/metagenome/mgm4440026.3"

]

],

"libraries": [

[

"mgl43388",

"http://api.metagenomics.anl.gov/1/library/mgl43388"

]

],

"created": "2010-07-01 00:00:00",

"env_package": {

"created": "2012-05-29 14:56:23",

"name": "mge43389",

"metadata": {

"medic_hist_perform": "yes",

"organism_count": "10^9 VLPs per ml",

"host_subject_id": "CFLungPat001Rep1",

"body_site": "lung",

"disease_stat": "non-exacerbated state",

"env_package": "human-associated",

"pulmonary_disord": "cystic fibrosis",

"sample_name": "mgs12326",

"body_product": "lung sputum"

},

"type": null,

"id": "mge43389"

},

"url": "http://api.metagenomics.anl.gov/1/sample/mgs12326",

"metadata": {

"geodetic_system": "wgs_84",

"elevation": "na ; Meter",

"collection_date": "2009-07-01",

"feature": "animal-associated habitat",

"latitude": "32.8781",

"biome": "animal-associated habitat",

"collection_timezone": "UTC",

"samp_collect_device": "Sputum samples of approximately 10 ml were obtained from CF patients at the Adult cystic fibrosis Clinic by expectoration into a sterile cup, as directed by clinic staff. Since sputum expectoration is difficult in general for Non-CF individuals, all Non-CF subjects were first required to do an oral rinse with water to prevent excessive salivary contamination and then take five deep breaths to loosen lung secretions. Subjects were then instructed to cough deeply into a sterile cup. The deep breathing and coughing procedures were repeated until at least 1 ml of sputum was obtained.",

"altitude": "na ; Meter",

"country": "United States of America",

"samp_size": "10 ml of sputum",

"longitude": "-117.1072",

"location": "Adult cystic fibrosis Clinic at the University of California San Diego Medical Center",

"isol_growth_condt": "19816605",

"depth": "na ; Meter",

"env_package": "human-associated",

"continent": "north_america",

"material": "animal-associated habitat"

},

"id": "mgs12326"

}
