# Supplementary material for: A RESTful API for Accessing Microbial Community Data for MG-RAST
Source: PLoS Comput Biol. 2015 Jan 8;11(1):e1004008. doi: 10.1371/journal.pcbi.1004008 (PMC4287624; doi:10.1371/journal.pcbi.1004008)
Supplement: S9 Example — A full-length example and abbreviated output for searching by metagenome ID. (DOCX) [file pcbi.1004008.s009.docx]

API call:

http://api.metagenomics.anl.gov/project/mgp31?verbosity=full

http://api.metagenomics.anl.gov/metagenome/mgm4440026.3?verbosity=full
