# Supplementary material for: A RESTful API for Accessing Microbial Community Data for MG-RAST
Source: PLoS Comput Biol. 2015 Jan 8;11(1):e1004008. doi: 10.1371/journal.pcbi.1004008 (PMC4287624; doi:10.1371/journal.pcbi.1004008)
Supplement: S10 Example — A full-length example and abbreviated output for searching metagenomes by metadata. (DOCX) [file pcbi.1004008.s010.docx]

API call:

http://api.metagenomics.anl.gov/metagenome?biome=marine&country=norway

Example cmd-line:

mg-search-metagenomes.py --biome marine --country norway

Example output:

mgm4440275.3 1-19-DNA-flx 2007-11-02T09:13:39Z public

mgm4440276.3 6-19-DNA-flx 2007-11-02T09:40:12Z public

mgm4440163.5 Bag1_13thMay_cDNA 2007-09-20T04:44:36Z public

mgm4440212.3 Bag1_13thMay_DNA 2007-10-03T08:50:15Z public

mgm4440164.6 Bag1_19thMay_cDNA 2007-09-28T06:17:52Z public
