# Supplementary material for: A RESTful API for Accessing Microbial Community Data for MG-RAST
Source: PLoS Comput Biol. 2015 Jan 8;11(1):e1004008. doi: 10.1371/journal.pcbi.1004008 (PMC4287624; doi:10.1371/journal.pcbi.1004008)
Supplement: S11 Example — A full-length example and abbreviated output for searching by function. (DOCX) [file pcbi.1004008.s011.docx]

API call:

http://api.metagenomics.anl.gov/metagenome?function=dnaA&order=name

Example cmd-line:

mg-search-metagenomes.py --function dnaA --order name

Example output:

mgm4440960.3 1229 1 mbsf ORIGINAL 2008-06-09T12:30:34Z public

mgm4440961.3 1229 1mbsf AMPLIFIED 2008-06-09T12:34:09Z public

mgm4440973.3 1229_16mbsf_AMPLIFIED 2008-06-10T09:25:31Z public

mgm4459940.3 1229_32mbsf_AMPLIFIED 2011-05-19T11:10:51Z public

mgm4459941.3 1229_50mbsf_AMPLIFIED 2011-05-19T11:10:51Z public
