# Supplementary material for: A RESTful API for Accessing Microbial Community Data for MG-RAST
Source: PLoS Comput Biol. 2015 Jan 8;11(1):e1004008. doi: 10.1371/journal.pcbi.1004008 (PMC4287624; doi:10.1371/journal.pcbi.1004008)
Supplement: S12 Example — A full-length example and abbreviated output for a complex search using function, metadata, and taxonomy. (DOCX) [file pcbi.1004008.s012.docx]

API call:

http://api.metagenomics.anl.gov/metagenome?function=dnaA&organism=coli&biome=marine&match=all&order=created

Example cmd-line:

mg-search-metagenomes.py --function dnaA --organism coli --order created --biome marine --match all

Example output:

mgm4440036.3 KingLIVir20050821 2007-05-21T18:37:44Z public

mgm4440037.3 KingLIMic20050821 2007-05-21T18:50:32Z public

mgm4440038.3 XmasLIVir20050805 2007-05-21T19:06:22Z public

mgm4440039.3 PalmLIMic20050818 2007-05-21T19:07:07Z public

mgm4440040.3 PalmLIVir20050818 2007-05-21T19:08:54Z public
