# Supplementary material for: A RESTful API for Accessing Microbial Community Data for MG-RAST
Source: PLoS Comput Biol. 2015 Jan 8;11(1):e1004008. doi: 10.1371/journal.pcbi.1004008 (PMC4287624; doi:10.1371/journal.pcbi.1004008)
Supplement: S13 Example — Access control. (DOCX) [file pcbi.1004008.s013.docx]

PERL

access_control.pl

# get a user agent

my $ua = LWP::UserAgent->new;

# set the authentication header

$ua->default_header('AUTH' => $auth_token);

# retrieve data requiring authentication

print $ua->get("<http://api.metagenomics.anl.gov/metagenome/mgm12345.3>")->content;

curl

curl -s -X GET -H "AUTH: myAuthTokenHere" "http://api.metagenomics.anl.gov/metagenome/mgm12345.3"

javascript

var xhr = new XMLHttpRequest();

xhr.open('GET',"http://api.metagenomics.anl.gov/metagenome/mgm12345.3");

xhr.setRequestHeader('AUTH', auth_token);

xhr.onload = function() { var metagenome = JSON.parse(xhr.responseText); }
