# Supplementary material for: A RESTful API for Accessing Microbial Community Data for MG-RAST
Source: PLoS Comput Biol. 2015 Jan 8;11(1):e1004008. doi: 10.1371/journal.pcbi.1004008 (PMC4287624; doi:10.1371/journal.pcbi.1004008)
Supplement: S14 Example — An example of metagenome as JSON object. (DOCX) [file pcbi.1004008.s014.docx]

{

**project**:

[

"mgp31",

"<http://api.metagenomics.anl.gov/project/mgp31>"

],

**version**: 1,

**migs**:

{

**country**: "United States of America",

**longitude**: "-117.1072",

**location**: "Adult cystic fibrosis Clinic at the University of California San Diego Medical Center",

**project**: "Human Lung Healthy vs Cystic Fibrosis Metagenome",

**sequence_type**: "WGS",

**seq_method**: "454",

**collection_date**: "2009-07-01 UTC",

**package**: "human-associated",

**feature**: "animal-associated habitat",

**latitude**: "32.8781",

**biome**: "animal-associated habitat",

**material**: "animal-associated habitat"

},

**status**: "public",

**name**: "CFLungPat001Rep1SDVir20060505",

**library**:

[

"mgl43388",

"<http://api.metagenomics.anl.gov/library/mgl43388>"

],

**sequence_type**: "WGS",

**created**: "2007-04-27 14:47:11",

**url**: "<http://api.metagenomics.anl.gov/metagenome/mgm4440026.3>",

**sample**:

[

"mgs12326",

"<http://api.metagenomics.anl.gov/sample/mgs12326>"

],

**id**: "mgm4440026.3",

**metadata**:

{

**env_package**:

{

**name**: "mge43389",

**type**: "human-associated",

**data**:

{

**disease_stat**: "non-exacerbated state",

**body_site**: "lung",

**env_package**: "human-associated",

**pulmonary_disord**: "cystic fibrosis",

**medic_hist_perform**: "yes",

**organism_count**: "10^9 VLPs per ml",

**host_subject_id**: "CFLungPat001Rep1",

**body_product**: "lung sputum"

},

**id**: "mge43389"

},

**project**:

{

**public**: "Human Lung Healthy vs Cystic Fibrosis Metagenome",

**name**: "Human Lung Healthy vs Cystic Fibrosis Metagenome",

**data**:

{

**project_funding**: "Cystic Foundation Research Inc. (www.cfri.org) ",

**firstname**: " - ",

**PI_organization**: "San Diego State University",

**PI_firstname**: "Dana",

**PI_organization_url**: "[http://sdsu.edu](http://sdsu.edu/)",

**lastname**: " - ",

**organization_url**: " - ",

**email**: " - ",

**PI_organization_address**: "San Diego, CA",

**PI_organization_country**: "USA",

**organization_address**: " - ",

**organization_country**: " - ",

**PI_lastname**: "Willner",

**ncbi_id**: "28593, 28441, 39545",

**project_description**: "The human respiratory tract is constantly exposed to a wide variety of viruses, microbes and inorganic particulates from environmental air, water and food. Physical characteristics of inhaled particles and airway mucosal immunity determine which viruses and microbes will persist in the airways. Here we present the first metagenomic study of DNA viral communities in the airways of diseased and non-diseased individuals. We obtained sequences from sputum DNA viral communities in 5 individuals with cystic fibrosis (CF) and 5 individuals without the disease.These results have important clinical implications for CF, indicating that therapeutic measures may be more effective if used to change the respiratory environment, as opposed to shifting the taxonomic composition of resident microbiota.",

**project_name**: "Human Lung Healthy vs Cystic Fibrosis Metagenome",

**PI_email**: "willner9@aol.com",

**organization**: " - "

},

**id**: "mgp31"

},

**library**:

{

**name**: "mgl43388",

**type**: "WGS",

**data**:

{

**metagenome_id**: "4440026.3",

**seq_meth**: "454",

**gold_id**: "Gm00009",

**metagenome_name**: "CFLungPat001Rep1SDVir20060505",

**sequences_sequences_filtered**: "Sequences with 80% identity over 80% of their length to human sequences were considered contaminating human genomic DNA and were removed prior to further bioinformatic analyses.",

**file_checksum**: "2c27a1acd82b4defa362ae987ed06094",

**pubmed_id**: "19816605, 18337718",

**investigation_type**: "metagenome"

},

**id**: "mgl43388"

},

**sample**:

{

**name**: "mgs12326",

**data**:

{

**geodetic_system**: "wgs_84",

**elevation**: "na ; Meter",

**collection_date**: "2009-07-01",

**feature**: "animal-associated habitat",

**latitude**: "32.8781",

**biome**: "animal-associated habitat",

**collection_timezone**: "UTC",

**samp_collect_device**: "Sputum samples of approximately 10 ml were obtained from CF patients at the Adult cystic fibrosis Clinic by expectoration into a sterile cup, as directed by clinic staff. Since sputum expectoration is difficult in general for Non-CF individuals, all Non-CF subjects were first required to do an oral rinse with water to prevent excessive salivary contamination and then take five deep breaths to loosen lung secretions. Subjects were then instructed to cough deeply into a sterile cup. The deep breathing and coughing procedures were repeated until at least 1 ml of sputum was obtained.",

**altitude**: "na ; Meter",

**country**: "United States of America",

**samp_size**: "10 ml of sputum",

**longitude**: "-117.1072",

**location**: "Adult cystic fibrosis Clinic at the University of California San Diego Medical Center",

**isol_growth_condt**: "19816605",

**depth**: "na ; Meter",

**env_package**: "human-associated",

**continent**: "north_america",

**material**: "animal-associated habitat"

},

**id**: "mgs12326"

}

}

}
